# Accumulation of antinuclear associated antibodies in circulating immune complexes is more prominent in SLE patients from Sudan than Sweden

**DOI:** 10.1038/s41598-020-78213-5

**Published:** 2020-12-03

**Authors:** Sahwa Elbagir, Azita Sohrabian, Amir I. Elshafie, Elnour M. Elagib, Nasr Eldeen A. Mohammed, Musa A. M. Nur, Elisabet Svenungsson, Iva Gunnarsson, Johan Rönnelid

**Affiliations:** 1 Department of Immunology, Genetics and Pathology, Uppsala University, Rudbeck Laboratory C5, 751 85 Uppsala, Sweden; 2 Rheumatology Unit, Alribat University Hospital, Khartoum, Sudan; 3 Rheumatology Unit, Military Hospital, Omdurman, Sudan; 4 Division of Rheumatology, Department of Medicine Solna, Karolinska Institutet, Karolinska University Hospital, Stockholm, Sweden; 5 Faculty of Medical Laboratory Sciences, Al Neelain University, Khartoum, Sudan

**Keywords:** Autoimmunity, Rheumatic diseases, Systemic lupus erythematosus

## Abstract

The role of anti-nuclear autoantibody (ANA) specificities in immune complexes (IC) formation has been studied to a limited extent in SLE, and not at all in African SLE patients. We compared ANA in IC from Sudanese and Swedish SLE patients. We included 93 Sudanese and 332 Swedish SLE patients fulfilling the 1982 ACR criteria. IC were captured using C1q-coated beads. ANA specificities were quantified in sera and IC. Results were related to modified SLEDAI. Whereas serum levels of anti-Sm, anti-dsDNA and anti-ribosomal P were higher in Swedish patients, IC levels of most ANA specificities were higher among Sudanese patients. This difference was especially prominent for anti-chromatin antibodies, which remained after adjustment for age, disease duration and treatment. Total levels of C1q-binding IC correlated with levels of specific ANA in IC, with highest correlations for anti-chromatin antibodies among Sudanese patients. Whereas occurrence of anti- SSA/Ro60, anti-histone and anti-U1RNP in both serum and IC associated with high SLEDAI score, anti-dsDNA in IC but not in serum associated with high SLEDAI. ANA, especially antibodies targeting chromatin, accumulate more in IC from Sudanese SLE patients. If the autoantibody fraction forming IC is pathogenically important, this might explain the generally described severe SLE in black populations.

## Introduction

Systemic lupus erythematosus (SLE) is an immune complex-mediated inflammatory condition with anti-nuclear autoantibodies (ANA) against nuclear-associated antigens. Among them, principally anti-double stranded DNA (dsDNA), are considered as hallmark of SLE. Immune complexes (IC) are formed by non-covalent binding of antibodies and corresponding antigen with or without the presence of complement proteins. IC clearance is mediated through complement or Fc receptors on immune cells, and defective removal can result in IC deposition and/or induction of inflammatory response thus causing tissue damage. IC containing RNA and DNA can bind to toll-like receptors 7 and 9 respectively, inducing plasmacytoid dendritic cell activation and interferon-α production ^1,2^.

Attempts to isolate and detect IC content in rheumatic diseases were documented decades ago. Different methodologies were utilized; the historic polyethylene glycol (PEG) precipitation technique, where immunogenic and non-immunogenic proteins are precipitated by mixing serum with low concentration of PEG followed by centrifugation^3^, was the most used. Despite sensitivity in isolating IC this method was confirmed non-specific^4,5^. Other techniques were also developed and modified to detect specific antigens like DNA or complement fragments contained in IC. Anti-dsDNA antibodies measurement before and after addition of DNAase buffer was reported to quantify levels of anti-dsDNA in IC^6^. Detection antibodies against C3 fragments^7^, the Raji cell assay in which sera are incubated with lymphoblastoid cell line to allow binding of IC to membrane C3 receptors^8^, C1q solid- and fluid-based assays^9–12^ were all used to capture and quantify circulating IC. Recently, a new technique was described to elute and quantify antigens bound to IC including monomeric IgG, using protein G and mass spectrometry^13^.

SLE is known to be prevalent and have more severe course with worse outcome among populations of African origin^14–16^. In our recently published paper comparing SLE in Sudan and Sweden, we have reported increased prevalence of anti-Sm antibodies with higher organ damage, markedly shorter disease duration and younger age among Sudanese patients suggesting reduced survival in that population^17^. Quantification of ANA in IC and association to SLE has not been studied in African populations, and previous studies showing association with disease activity involved mainly non-African ethnicities^7,18^.

Our aim in the current study was to compare ANA levels in purified IC from Sudanese and Swedish SLE patients’ sera and to relate this to distribution of corresponding serum levels in the two countries. We have used a novel assay developed and validated by our group, utilizing C1q-coated beads to capture IC from patients’ sera^19^. The technique was used earlier to quantify anti-citrullinated peptide antibodies in IC found in serum and synovial fluid of Swedish rheumatoid arthritis patients^19^, and in another study to evaluate response to belimumab treatment in SLE^18^.

## Methods

### Patients

Ninety-three consecutive Sudanese and 332 Swedish SLE patients classified according to the 1982 revised (ACR) criteria^20^, were included in this study. Complete description of these cohorts was detailed in a previous publication^17^. Information about demographics, ongoing treatment and scores for Systemic Lupus Erythematosus Disease Activity Index (SLEDAI)^21^, was acquired for Sudanese and Swedish patients at time of study inclusion. To compare the cohorts, SLEDAI was modified by excluding urinary parameters, DNA binding and complement components due to incomplete data in the Sudanese cohort. Serum samples for immunological analyses were available for all participants. Demographic data, SLEDAI score and ongoing treatment are outlined in Table 1.

### Immunological testing

Circulating IC were purified from patients’ sera using magnetic micro-particles coated with purified human C1q. For capturing and elution of IC, 20 μL of C1q-coated beads are incubated with 10 μL patient’s serum in a 96-well plate on microplate shaker for 1.5 h. Thereafter, beads with C1q-bound IC were recovered on a magnetic washer. After washing with PBS-0.05% tween, IC were eluted in a two-step procedure as previously described^19^, using two buffers at different pH, in sequence, to ensure effective elution. The eluted IC were then stored at − 70 °C until measurement.

Quantification of ANA dsDNA, Sm, the Sm/U1RNP complex, U1RNP, SSA/Ro52, SSA/Ro60, SSB/La, ribosomal P antigen, proliferating cell nuclear antigen (PCNA) and histones was performed in sera and in the recovered eluates containing solubilized IC using an addressable laser bead immunoassay, (FIDIS connective tissue profile, Theradiag, Marne la Vallee, France). Positivity for specific ANA in serum was determined using manufacturer’s suggested cutoff (40 U/ml). Cutoffs for ANA in IC were calculated based on correlations between levels of ANA in serum and corresponding IC and related to the manufacturer’s serum cutoff in a linear regression formula. Circulating C1q-binding IC (CIC) were measured using the Quanta Lite CIC ELISA (Inova Diagnostics, San Diego, CA). CIC data in Sudanese and Swedish SLE patients were previously published^17^, here only used for correlations with ANA in IC. Serum levels of antibodies have also been described previously, where the number of Swedish patients were slightly higher (n = 337)^17^. Isolation and detection of ANA in IC were performed at a later time point. All ANA measurements in serum and in IC were done in parallel for Sudanese and Swedish patients.

### Statistical analysis

For comparisons between quantitative variables, Mann–Whitney’s U test was used, and to adjust for confounding factors multiple linear regression analyses were applied. Chi^2^-test was used to test for differences between categorical variables with Fisher’s exact test applied when appropriate. Spearman’s non-parametric correlation test was used to measure strength and direction of correlation between quantitative variables. All statistical analyses were conducted using JMP statistical software (SAS institute, Cary, NC, USA). P values < 0.05 were considered significant.

### Ethics approval and consent to participate

All patients gave written informed consent and the study conformed to the guidelines of Declaration of Helsinki. The Ethics Committees of Alribat University hospital, Khartoum, Sudan and Omdurman Military hospital, Omdurman, Sudan gave approval for the Sudanese cohort/study (11 April 2011 and 25 May 2011, respectively) and the Uppsala and Karolinska University Hospital Ethics Committees approved the Swedish cohort/study (03-556 [16 Dec 2003]).

## Results

### Levels and occurrence of specific ANA in sera and IC

**Figure 1:**
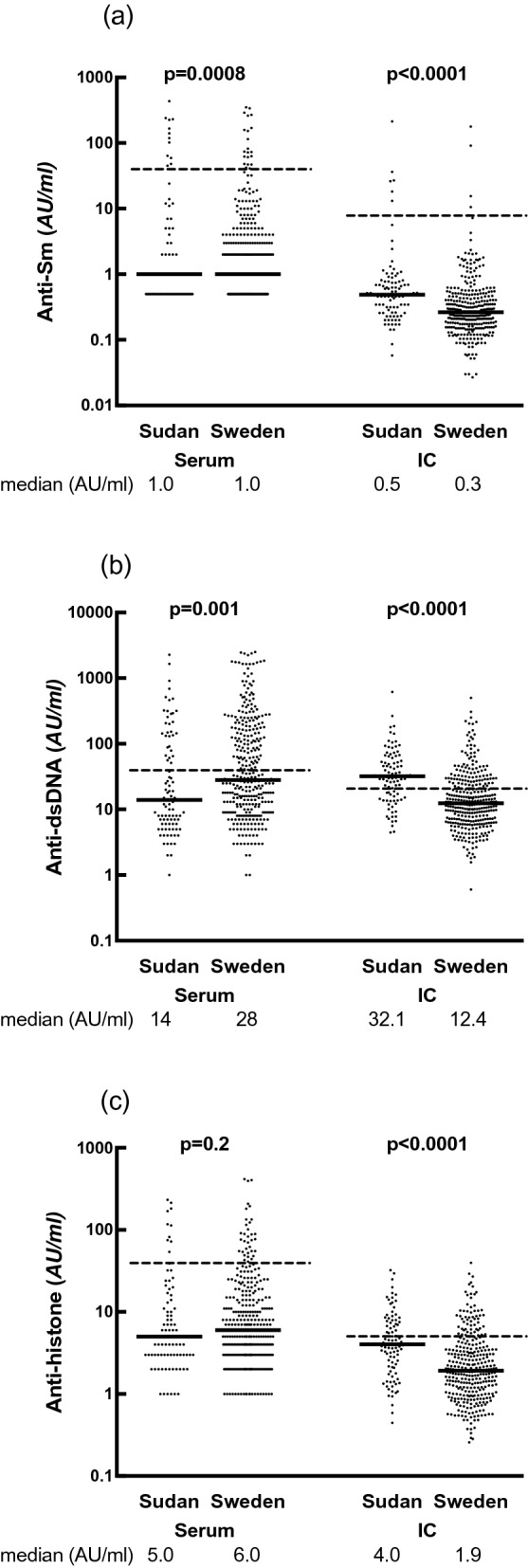
Anti-nuclear autoantibody specificities in serum and immune complexes (IC). Median levels of **(a)** anti-Sm, **(b)** anti-dsDNA and **(c)** anti-histone autoantibodies in serum and in corresponding IC among Sudanese and Swedish SLE patients. Horizontal dashed lines represent cutoffs for levels in serum and IC.

Sudanese patients had lower serum levels than Swedish patients of anti-Sm (median/interquartile range, 1.0/0–3.5 vs. 1.0/1.0–4.0; P = 0.0008), anti-dsDNA (14.0/6.0–110.5 vs. 28.0/11.0–166.0; p = 0.001) and anti-ribosomal P antigen (1.0/1.0–3.0 vs. 2.0/1.0–5.0; p = 0.0006). However, this situation was completely reversed for levels of specific ANA in solubilized IC from the same serum samples; levels of all antibodies except anti-SSA/Ro52 and anti-SSA/Ro60 were higher in IC from Sudanese than from Swedish patients. This difference was highly significant for most ANA specificities, with more than twofold difference in median levels of anti-chromatin antibodies detected in IC from Sudanese patients (anti-dsDNA; 32.1/15.5–57.7 vs. 12.4/6.8–25.9; p < 0.0001 and anti-histone; 4.0/2.1–7.6 vs. 1.9/1.0–3.5; p < 0.0001), Table 2 and Fig. 1. After adjustment for age and disease duration at study inclusion in multiple linear regressions, higher levels of anti-dsDNA (standardized β = 0.12, p = 0.02) and anti-histone (standardized β = 0.14, p = 0.008) among the Sudanese patients remained, whereas significances for other ANA specificities were lost. Moreover, when we added the use of DMARDS, hydroxychloroquine and prednisolone to age and disease duration all as independent variables, significance for anti-dsDNA and anti-histone also remained (standardized β = 0.11/p = 0.04 and standardized β = 0.15/p = 0.009 respectively).

**Table 1:** Demographics and ongoing treatment in 93 Sudanese and 332 Swedish SLE patients.

Demographics and treatment	Sudan	Sweden	P value
Gender (F%)	96.8%	88.1%	**0.01**
Age at inclusion (median/mean) Years	35/36	47.7/46.8	** < 0.0001**
Duration of disease (median/mean) Years	4/4.9	15/17.9	** < 0.0001**
SLEDAI score (median/mean)	0/1.7	0/1.7	0.4
Hydroxychloroquine n (%)	49 (54.4%)	113 (34.6%)	**0.0006**
Prednisolone n (%)	61 (67.8%)	188 (57.1%)	0.06
Prednisolone dose (median/mean) mg	5.0/10.0	2.5/5	**0.01**
Any DMARDS at inclusion n (%)	73 (81.1%)	105 (35.6%)	** < 0.0001**
Azathioprine n (%)	59 (65.6%)	56 (17.2%)	** < 0.0001**
Mycophenolate mofetil n (%)	12 (13.3%)	32 (9.9%)	0.3
Methotrexate n (%)	8 (8.9%)	14 (4.3%)	0.08
Cyclophosphamide n (%)	2 (2.2%)	4 (1.4%)	0.6
Cyclosporine n (%)	1(1.1%)	2 (0.6%)	0.6

Significant P values are depicted in bold and underlined to indicate higher prevalence in Sudanese patients.

*DMARDS* disease modifying anti-rheumatic drugs (azathioprine, mycophenolate mofetil, methotrexate, cyclophosphamide and cyclosporine), *F* female.

We then performed separate regressions for each of the demographical parameters and pharmacological treatments, adjusted for country. Older age and long disease duration were both associated with lower IC levels of anti-dsDNA (standardized β = − 0.11/p = 0.03 and standardized β = − 0.14/p = 0.006), anti-histone (standardized β = − 0.12/p = 0.01 and standardized β = − 0.13/p = 0.01), anti-Sm/U1RNP (standardized β = − 0.14/p = 0.005 and − 0.18/p = 0.0008) and anti-U1RNP (standardized β = − 0.16/p = 0.001 and − 0.16/p = 0.003). In addition, lower IC levels of anti-SSB (standardized β = − 0.13/p = 0.01) and anti-PCNA (standardized β = − 0.11/p = 0.047) also associated with longer disease duration. The use of hydroxychloroquine was not associated with any ANA in IC, while prednisolone usage associated with increased levels of anti-dsDNA (standardized β = 0.12/p = 0.01), anti-histone antibodies (standardized β = 0.11/p = 0.02), anti-Sm/U1RNP (standardized β = 0.11/p = 0.02), anti-U1RNP (standardized β = 0.13/p = 0.008) and anti-PCNA (standardized β = 0.12/p = 0.01) in IC. Effects were stronger in the larger Swedish cohort.

Occurrence of specific ANA in serum did not differ between Sudanese and Swedish patients, except for higher prevalence of anti-Sm in Sudan (p = 0.04). Occurrence of antibodies in solubilized IC was on the other hand more common among Sudanese compared to Swedish patients for antibodies against Sm, Sm/U1RNP, dsDNA and histone. Also, this difference was especially prominent for anti-dsDNA and anti-histone in IC from Sudanese compared to Swedish SLE patients (p < 0.0001 for both; Table 3).

**Table 2:** Levels (median/interquartile range IQR) of antinuclear-associated autoantibodies in serum and in corresponding Immune complexes (IC).

ANA	Serum			IC		
	Sudan, median/IQR	Sweden, median/IQR	P	Sudan, median/IQR	Sweden, median/IQR	P
SSA/Ro52	16.0/10.0–75.5	17.0/9.0–70.0	0.8	2.9/1.5–9.8	2.4/1.2–6.0	0.07
SSA/Ro60	2.0/0–106.0	3.0/1.0–111.0	0.2	1.5/0.6–20.2	1.3/0.5–15.3	0.7
SSB/La	2.0/1.0–13.0	2.0/1.0–15.0	0.6	1.1/0.6–1.9	0.8/0.4–1.9	**0.02**
Sm	1.0/0–3.5	1.0/1.0–4.0	**0.0008**	0.5/0.2–0.8	0.3/0.2–0.4	** < 0.0001**
Sm/U1RNP	1.0/0–10.0	1.0/1.0–10.0	0.2	3.7/1.8–7.2	2.0/1.2–4.0	** < 0.0001**
U1RNP	6.0/3.0–25.5	7.0/3.0–27.0	0.9	1.0/0.6–2.4	0.6/0.4–1.4	**0.0005**
dsDNA	14.0/6.0–110.5	28.0/11.0–166.0	**0.001**	32.1/15.5–57.7	12.4/6.8–25.9	** < 0.0001**
Histone	5.0/3.0–12.0	6.0/3.0–15.0	0.2	4.0/2.1–7.6	1.9/1.0–3.5	** < 0.0001**
Ribosomal P	1.0/0.6–3.0	2.0/1.0–5.0	**0.0006**	1.7/1.0–3.0	1.4/0.8–2.2	**0.005**
PCNA	5.0/3.0–10.5	5.0/3.0–10.0	0.6	11.1/6.7–18.9	7.0/4.0–12.4	** < 0.0001**

Significant P values are depicted in bold and underlined to indicate higher levels in Sudanese patients.

### Correlation between total levels of circulating C1q-binding IC and levels of specific ANA in IC

Total levels of circulating CIC in serum correlated with levels of most individual ANA specificities in purified IC from the same serum samples, except for anti-SSA/Ro52 and anti-PCNA. For all investigated ANA specificities, the corresponding correlation coefficients were higher for the Sudanese than for the Swedish SLE patients. Although the degrees of correlation were rather low, they were highest for anti-chromatin antibodies directed against dsDNA (Sudanese patients: ρ = 0.36, p = 0.0004 and Swedish patients: ρ = 0.35, p < 0.0001) and histone (Sudanese patients: ρ = 0.38, p = 0.0002 and Swedish patients: ρ = 0.18, p = 0.001; Table 4).

**Table 3:** Prevalence of antinuclear-associated autoantibodies in serum and in corresponding immune complexes (IC).

ANA	Serum			IC		
	Sudan, n (%)	Sweden, n (%)	P	Sudan, n (%)	Sweden, n (%)	P
SSA/Ro52	30(32.2)	105(31.7)	0.9	18(19.3)	55(16.6)	0.5
SSA/Ro60	34(36.6)	122(36.9)	0.9	27(29)	89(26.8)	0.7
SSB/La	15(16.1)	64(19.3)	0.5	9(9.7)	38(11.4)	0.3
Sm	12(12.9)	21(6.3)	**0.04**	6(6.4)	5(1.5)	**0.008**
Sm/U1RNP	15(16.1)	42(12.7)	0.4	17(18.3)	33(9.9)	**0.03**
U1RNP	20(21.5)	69(20.8)	0.9	22(23.7)	55(16.6)	0.1
dsDNA	31(33.3)	147(44.4)	0.055	64(68.8)	105(31.6)	** < 0.0001**
Histone	10(10.7)	34(10.3)	0.9	34(36.6)	58(17.5)	** < 0.0001**
Ribosomal P	4(4.3)	20(6)	0.5	2(2.1)	9(2.7)	0.8
PCNA	1(1.1)	10(3)	0.3	20(21.5)	45(13.5)	0.06

Significant P values are depicted in bold and underlined to indicate higher levels in Sudanese patients.

### Association between disease activity and specific ANA in serum and IC

Whereas occurrence of antibodies against Sm and the Sm/U1RNP complex in serum associated with high SLEDAI scores among Sudanese patients, anti-U1RNP in both serum and in IC associated with high disease activity. More ANA specificities in IC than in serum associated high SLEDAI scores among Swedish patients; associations to anti-U1RNP and anti-dsDNA were only found for IC bound antibodies (Table 5). Occurrence of ANA was never associated with low SLEDAI scores.

**Table 4:** Correlations between C1q-binding immune complexes (CIC) and levels of antinuclear antibody (ANA) specificities detected in IC.

ANA in IC	Sudan Correlation to CIC ρ (P)	Sweden Correlation to CIC ρ (P)
SSA/Ro52	0.15 (0.1)	0.004 (0.9)
SSA/Ro60	**0.21 (0.04)**	0.04 (0.5)
SSB/La	**0.23 (0.02)**	0.002 (0.9)
Sm	**0.26 (0.01)**	**0.17 (0.001)**
Sm/U1RNP	**0.20 (0.048)**	**0.13 (0.01)**
U1RNP	**0.26 (0.01)**	**0.14 (0.008)**
dsDNA	**0.36 (0.0004)**	**0.35 (< 0.0001)**
Histone	**0.38 (0.0002)**	**0.18 (0.001)**
Ribosomal P	0.17 (0.1)	**0.09 (0.001)**
PCNA	0.15 (0.1)	0.04 (0.5)

Significant P values and corresponding correlation coefficients are depicted in bold.

**Table 5:** Modified SLEDAI scores in relation to occurrence of individual ANA specificities in sera and IC.

	Serum				IC			
ANA	SLEDAI median in antibody neg/pos patients Sudan	P	SLEDAI median in antibody neg/pos patients Sweden	P	SLEDAI median in antibody neg/pos patients Sudan	P	SLEDAI median in antibody neg/pos patients Sweden	P
SSA/Ro52	0/0	0.3	0/1	0.07	0/0	0.6	0/1	0.08
SSA/Ro60	0/0	0.2	0/1	**0.007**	0/0	0.5	0/1	**0.02**
SSB/La	0/1	0.2	0/0	0.2	0/0	0.9	0/0	0.6
Sm	0/3	**0.0007**	0/1	0.06	0/2	0.08	0/1	0.5
SmRNP	0/2	**0.004**	0/1	0.1	0/0	0.2	0/1	0.08
U1RNP	0/2	**0.001**	0/1	0.1	0/2	**0.001**	0/1	**0.002**
dsDNA	0/0	0.1	0/0	0.2	0/0	0.7	0/1	**0.02**
Histone	0/0	0.2	0/1	**0.01**	0/0	0.1	0/2	** < 0.0001**
Ribosomal P	0/2	0.3	0/1	0.1	0/0	0.3	0/0	0.9
PCNA	0/0	0.5	0/1	0.2	0/0	0.8	0/1	0.06

Data are expressed as median score of SLEDAI among autoantibody negative vs. positive patients. Significant P values are depicted in bold.

## Discussion

Using a novel IC purification technique, we have shown that specific ANA accumulate more in circulating IC of Sudanese compared to Swedish SLE patients; a finding opposite to the distribution for the corresponding serum levels. Also, occurrence of many ANA was more common in IC from Sudanese patients. Whether this accumulation of ANA in IC in Sudanese patients depends on genetic or environmental factors, we do not know. It is however probably not secondary to generally higher IC levels in Sudan, as we previously have shown that levels of CIC were higher in the investigated Swedish than in the Sudanese SLE cohort^17^.

In a South African study by Pudifin D et al., healthy black subjects had increased circulating IC using Raji cell assay compared to whites and Indians^22^. In the current study, ANA in IC were not evaluated in healthy controls, thus we could not determine whether the difference in ANA IC between Sudanese and Swedish patients could be attributed to a general increase in the Sudanese healthy population. Interestingly, we found that Sudanese SLE patients have higher IgA anti-phospholipid antibodies compared to Swedes, but this difference disappeared after adjustment to national controls, as levels were higher also among Sudanese than Swedish healthy subjects^23^. Optimally, IC levels as well as levels of autoantibodies in IC, should in the same way be related to national controls before comparison between patient groups from different geographical regions^17^.

After adjustment for the difference in age and disease duration between Sudanese and Swedish patients that we described previously^17^, the increase detected in IC among Sudanese patients remained significant for anti-histone and anti-dsDNA. Interestingly, total circulating CIC levels correlated also best with levels of anti-histone and anti-dsDNA antibodies in IC in Sudanese patients, indicating that anti-chromatin or anti-dsDNA with or without chromatin contamination might constitute an important part of IC content, especially in Sudanese patients. It is noteworthy that IC containing antibodies against complexed chromatin structures were previously shown to be the main pathogenic IC in SLE nephritis^24–26^, therefore, further investigation of presence and molecular size of DNA-related antigens within these IC might explain the accumulation of anti-chromatin antibodies in IC from Sudanese patients. Moreover, studies investigating antigenic constituents and molecular size of IC would add knowledge to the understanding of IC in populations where tropical infections prevail^27,28^.

In a previous study on belimumab-treated Swedish SLE patients using the current technique we also found that levels of anti-dsDNA in IC correlated more strongly to total circulating CIC levels than any other ANA specificity^18^.

More Sudanese patients were on hydroxychloroquine, which is a reasonably available drug in Sudan, and were also treated with higher doses of prednisolone. The use of higher prednisolone doses is likely to reflect active disease at the time the drug was prescribed; this might explain the association of prednisolone with higher levels of ANA in IC. But interestingly, corticosteroids have also previously been reported to increase circulation IC levels, at least for a short-term^29^.

Disease activity among Swedish SLE patients was associated with more ANA specificities in IC than in serum, showing associations with anti-U1RNP and anti-dsDNA in IC but not in serum. Anti-U1RNP in IC was found to associate with higher SLEDAI both in Sudan and Sweden. It has previously been shown that IC containing U1RNP antibodies activate plasmacytoid dendritic cells and induce production of interferon-α through toll-like receptor 7, promoting further inflammation and immune cell activation^2,30,31^. On the other side, several studies failed to find associations between anti-U1RNP IC and SLE activity, however, interpretation of these studies might be affected by the fact that different IC isolation techniques that are known to co-precipitate high-molecular weight proteins in the serum sample were used^7,27^. A follow up investigation of the role of interferon-α in Sudanese vs. Swedish SLE patients in relation to ANA content in IC and disease activity would therefore be interesting.

Possible technical limitations in our study is the co-purification of anti-C1q antibodies which bind to the collagenous part of C1q, and that IC already covered by activated complement might bind poorly or not at all to the C1q-coated beads. Serum ANA investigations had been performed previously^17^ and quantification of ANA in IC was performed later with different reagent batches. For practical reasons, eluted IC had been stored frozen at − 70 °C and not quantified immediately upon IC purification, as performed previously^18^.

We deem that the comparison between the Sudanese and Swedish cohorts concerning levels of ANA in serum and in IC respectively are well-founded since for both serum and IC, samples from both countries were analyzed in parallel.

To our knowledge, this study is the first ever to quantify ANA in circulating IC in SLE patients from Africa. The propensity of ANA accumulation in IC from Sudanese patients could be related to ethnic/environmental factors with generally high IC levels in black populations^22^. More investigations are planned to uncover possible underlying causes. In future studies we will relate immune-associated single nucleotide polymorphisms including imputed human leukocyte antigen alleles to ANA levels in serum and IC respectively.

## Data Availability

All data supporting our findings are included in the article.

## Abbreviations

ACR: American College of Rheumatology
ANA: Anti-nuclear associated antibodies
CIC: C1q-binding immune complexes
DMARDS: Disease-modifying anti-rheumatic drugs
DsDNA: Double stranded DNA
IC: Immune complexes
PCNA: Proliferating cell nuclear antigen
SLE: Systemic lupus erythematosus
SLEDAI: Systemic lupus erythematosus disease activity index
